# Factors influencing postoperative outcomes in patients with symptomatic discoid lateral meniscus

**DOI:** 10.1186/s12891-020-03573-y

**Published:** 2020-08-15

**Authors:** Shun-Jie Yang, Zhong-Jun Ding, Jian Li, Yang Xue, Gang Chen

**Affiliations:** 1 Department of Orthopedic Surgery, West China Hospital, Sichuan University, No.37, Guoxue Alley, Chengdu, 610041 China; 2 Department of Orthopedic Surgery, West China Longquan Hospital Sichuan University, No.201, Yihe Group 3, Longquanyi District, Chengdu, 610100 China

**Keywords:** Influential factor, Discoid meniscus, Arthroscopy, Clinical effect

## Abstract

**Background:**

Due to its abnormal morphology and ultrastructure, discoid lateral meniscus (DLM) is prone to tear and degeneration, leading to clinical symptoms. Arthroscopy is the main treatment for symptomatic DLM; however, postoperative outcomes vary widely due to the effects of diverse factors. This research aims to explore the factors influencing postoperative outcomes of symptomatic DLM.

**Methods:**

Patients with DLM who underwent arthroscopic surgery at our hospital from 9/2008 to 9/2015 were enrolled according to the inclusion and exclusion criteria. Fourteen variables, including sex, body mass index (BMI) and other variables, were chosen as factors for study. Knee function was assessed using the International Knee Documentation Committee (IKDC) score. Univariate analyses (Mann-Whitney U test or Kruskall-Wallis rank sum test) and multivariate analyses (ordinal logistic regression) were used to identify the factors that influenced postoperative outcomes. *P* < 0.05 was considered statistically significant.

**Results:**

A total of 502 patients, including 353 females (70.3%) and 149 males (29.7%), were enrolled. The median IKDC score postoperatively (87.4; range, 41.4 ~ 97.7; IQR, 14.6) was higher than that preoperatively (57.6; range, 26.9 ~ 64.9; IQR, 9.7) (*P* < 0.001). Male sex was predictive of a higher IKDC score (*P* = 0.023, OR = 1.702). Compared with BMI ≥25 kg/m^2^, *< 18.5 kg/m*^*2*^ was associated with better IKDC score (*P* = 0.026, OR = 3.016). Contrasting with age of onset ≥45 years, *≤14 years* (*P* < 0.001, OR = 20.780) and *14 ~ 25 years* (*P <* 0.001, OR = 8.516) were associated with better IKDC score. In comparison with symptoms duration> 24 months, IKDC scores for patients with symptoms duration *≤1 month* (*P* = 0.001, OR = 3.511), *1 ~ 6 months* (*P* < 0.001, OR = 3.463) and *6 ~ 24 months* (*P* < 0.001, OR = 3.254) were significantly elevated. Compared to Outerbridge grade III ~ IV, no injury (*P <* 0.001, OR = 6.379) and grade I (*P* = 0.01, OR = 4.332) were associated with higher IKDC score.

**Conclusions:**

Arthroscopic treatment of symptomatic DLM is safe and effective, but its clinical efficacy is affected by many factors. Specifically, male sex, BMI < 18.5 kg/m^2^, age of onset < 25 years (especially < 14 years) and symptoms duration < 24 months are conducive to good postoperative outcomes. However, combined articular cartilage injury (Outbridge grade ≥ 2) reduces postoperative effect.

## Background

The discoid meniscus is a congenital variation of the knee meniscus. Morphologically, the discoid meniscus is wider and thicker than the normal meniscus, resulting in potential instability. In the ultrastructure of the discoid meniscus, the radial and circular collagen fibre systems are reduced and disorganized, and the circular collagen fibres are insufficient in strength and show increased brittleness. These morphological and histological abnormalities make the discoid meniscus liable to tearing and degeneration [1–3].

Clinically, discoid lateral meniscus (DLM) is common. The reported incidence of DLM is 13% in china [4], 16.6% in Japan [5], 10.9% in Korea [6] and 5.8% in India [7]. The prevalence of DLM in the western population ranges from 0.4 to 17% [8]. Patients suffering from bilateral DLM accounts for 79–97% of cases [9].

Symptomatic DLM, characterized by pain, swelling, snapping, locking, knee instability and limited mobility [1, 10], is mainly diagnosed by magnetic resonance imaging (MRI) and treated by arthroscopic surgery [11]. Although the overall postoperative clinical outcomes of symptomatic DLM are acceptable [12–14], the outcomes still differ among individuals, possibly as a result of the diversity in patient characteristics and treatments [11]. Recently studies have reported several factors that influence postoperative effect in symptomatic DLM, but the sample sizes used in these studies are uneven, and the results are inconsistent [12, 15–17]. The present research independently analyses 14 possible influences, including gender, body mass index (BMI), and other factors, in a larger series of patients with the goal of identifying the factors that influence postoperative outcome in patients with symptomatic DLM and thereby guiding clinical application.

## Methods

### Subjects

**Fig. 1:**
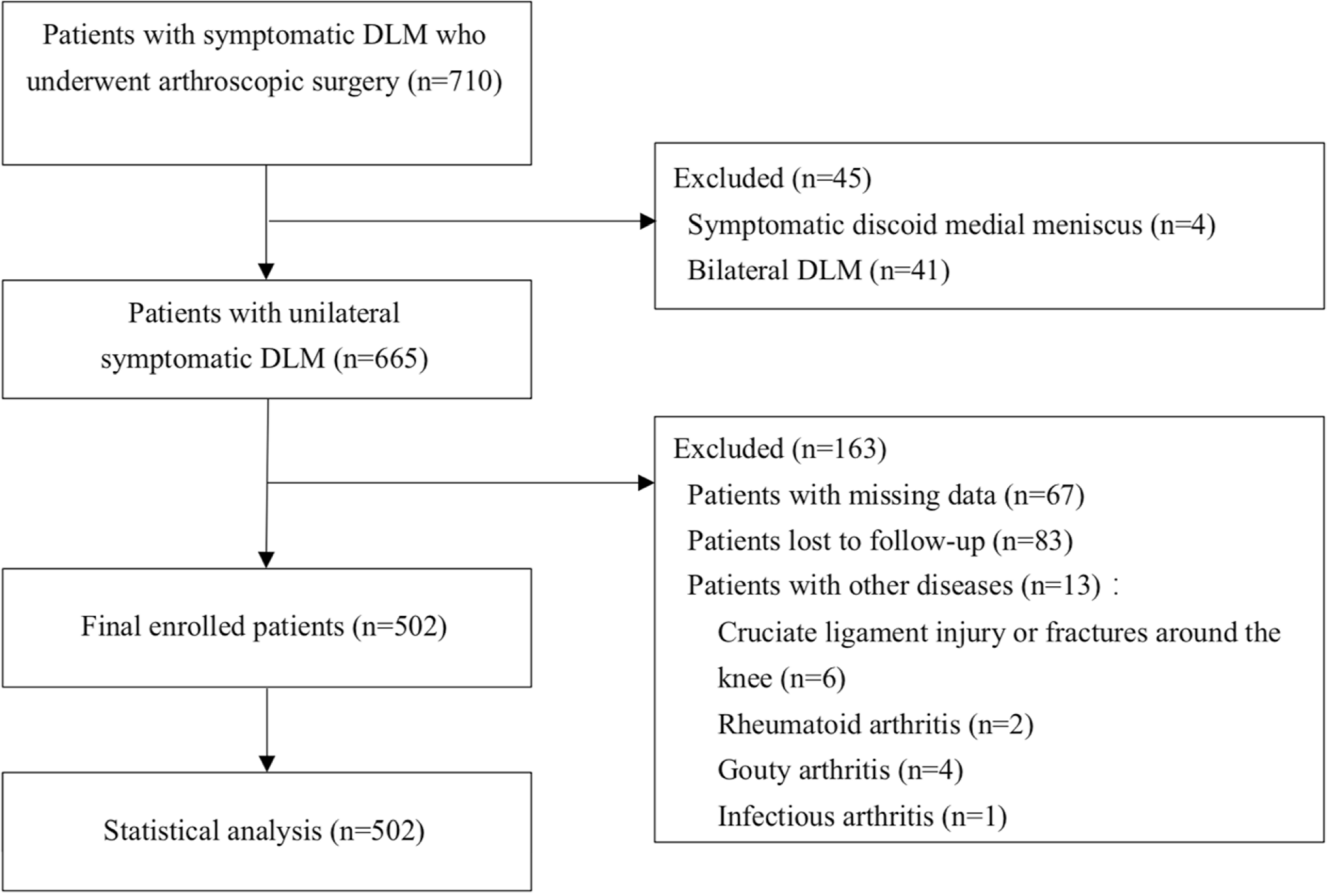
Flow chart of the study

This study was approved by the institutional review board of our institution. The inclusion criteria were as follows: DLM patients with symptoms (pain, swelling, snapping, locking, knee instability and limited mobility) who underwent arthroscopic surgery at the Sports Medicine Center of West China Hospital of Sichuan University from September 2008 to September 2015. The exclusion criteria were as follows: patients with missing data; patients lost to follow-up; patients with bilateral symptomatic DLM who underwent bilateral knee operation; patients with symptomatic discoid medial meniscus, knee ligament injury or knee fracture; patients with rheumatoid arthritis or gouty arthritis of the knee; and patients with knee infections. A flow chart of the study is shown in Fig. 1.

### Research methods

The following 14 independently factors that might possibly affect the postoperative results of patients with symptomatic DLM were collected from the patients’ medical records, imaging data and arthroscopy videos: sex; BMI (kg/m^2^); work intensity (based on‘REFA daily life and work intensity classification’ [18], schedule 1); trauma history (clinical symptoms of knee joints due to sports, sprains, falls, etc.); involved knee joint (left or right); age of onset; duration of symptoms; DLM type according to the Watanabe Classification [17], schedule 2; type of DLM tear (based on the O’Connor classification of meniscus tears [19], schedule 3); medial meniscus tear; severity of cartilage lesion classified according to Outerbridge grade [20], schedule 4; Kellgren-Lawrence classification of osteoarthritis grade [21] (K-L grade, schedule 5); method of surgery (saucerization, saucerization with repair, total meniscectomy); and final follow-up time. The intraarticular factors listed above, liking DLM type, Outerbridge grade, etc. were evaluated by arthroscopy. The choice of surgical mode depends primarily on the condition of the DLM. If the circular fibres of the DLM are continuous, saucerization is used; if they are discontinuous, total meniscectomy is conducted; if the DLM is continuous and accompanied by instability or tear of a peripheral rim or a repairable tear in the red zone, saucerization with repair is performed. All of the surgical procedures for all patients were performed by the same senior surgeon, whose surgical technique was reliable and stable. Preoperative and postoperative knee function were evaluated using the IKDC subjective knee evaluation form [22] (schedule 6). The score was obtained through periodic outpatient follow-up. In the overall scores of IKDC, scores of 90–100, 80–89, 70–79 and <  70 points were categorized as “excellent,” “good,” “fair,” and “poor,” respectively [22]. Given the clinical application, we combined fair and poor into one level (fair-poor) representing an unsatisfactory postoperative effect. The basic characteristics of the included subjects are shown in Table 1. The assignment of categorical variables and the IKDC classification are shown in Table 2. The factors affecting the postoperative outcomes of symptomatic DLM were explored by analysing the correlation between the above 14 factors and IKDC classification.

**Table 1:** Basic characteristics of the included patients

Demographic details	N (%)/M (range; IQR) †	Demographic details	N (%)/M (range; IQR) †	Demographic details	N (%)/M (range; IQR) †
**Patients**	502	≤14	88(17.5%)	Yes	18 (3.6%)
**Sex**		14–25	101(20.1%)	**Outerbridge grade**	
Male	149 (29.7%)	25–45	202(40.2%)	No lesion	358 (71.3%)
Female	353 (70.3%)	≥45	111(22.1%)	Grade I	29 (5.8%)
**BMI** (kg/m^2^)	22.0 (range, 13.8 ~ 44.4; 4.3)	**Symptoms duration** (months)	10.0 (range, 0.05 ~ 246; 21.0)	Grade II	52 (10.4%)
< 18.5	54(10.8%)	≤1	64(12.7%)	Grade III	23 (4.6%)
18.5–25	362(72.1%)	1–6	149(29.7%)	Grade IV	40 (8.0%)
≥25	86(17.1%)	6–24	184(36.7%)	**K-L grade**	
**Work intensity**		>24	105(20.9%)	Grade 0	375 (74.7%)
Grade 0	27 (5.6%)	**Watanabe type**		Grade I	56 (11.2%)
Grade 1	212 (42.2%)	Complete	423 (84.3%)	Grade II	44 (8.8%)
Grade 2	240 (47.8%)	Incomplete	79 (15.7%)	Grade III	23 (4.6%)
Grade 3	22 (4.4%)	**O’Connor type**		Grade IV	4 (0.8%)
Grade 4	1 (0.2%)	No tear	38 (7.6%)	**Type of surgery**	
**Trauma history**		Longitudinal (/bucket handle) tear	171 (34.1%)	Saucerization	410 (81.7%)
No	360 (71.7%)	Horizontal tear	154 (30.7%)	Saucerization with repair	16 (3.2%)
Yes	142 (28.3%)	Oblique tear	10 (2.0%)	Ttotal meniscectomy	76 (15.1%)
**Involved knee joint**		Transverse (/radial) tear	45 (9.0%)	**Follow-up time** (months)	75.4 (range, 41.0 ~ 123.3; 33.7)
Left	252 (50.2%)	Variant tear	84 (16.7%)	≤60	135(26.9%)
Right	250 (49.8%)	**Medial meniscus tear**		60–96	254(50.6%)
**Age of onset** (years)	32.0 (range, 3 ~ 80; 26.3)	No	484 (96.4%)	>96	113(22.5%)

*BMI* body mass index, *K-L grade* Kellgren–Lawrence grade, *DLM* discoid lateral meniscus, *IKDC* International Knee Documentation Committee, † *N* number, *M* median, *IQR* interquartile range

**Table 2:** Assignment of research factors and IKDC classification

Factors/IKDC classification	Valuation
Sex (X_1_)	male “0”; female “1”
BMI (kg/m^2^) (X_2_)	X_2_ < 18.5 “1”; 18.5 ≤ X_2_ < 25 “2”; X_2_ ≥ 25 “3”
Work intensity (X_3_)	grade 0 ~ 1 “1”; grade 2 “2”; grade 3 ~ 4 “3”
History of trauma (X_4_)	no “0”; yes “1”
Involved knee joint (X_5_)	left knee “0”; right knee “1”
Age of onset (year) (X_6_)	X_6_ ≤ 14 “1”; 14 < X_6<_ 25 “2”; 25 ≤ X_6_ < 45 “3”; X_6_ ≥ 45 “4”
Symptom duration (month) (X_7_)	X_7_ ≤ 1 “1”; 1 < X_7_ ≤ 6 “2”; 6 < X_7_ ≤ 24 “3”; X_7_ > 24 “4”
Watanabe type (X_8_)	type I (complete) “1”; type II (incomplete) “2”
O’Connor type (X_9_)	no “0”; longitudinal (/bucket handle) tear “1”; horizontal tear “2”; oblique tear “3”; transverse (/radiation) tear “4”; variant tear (including flap, composite, degenerate meniscus tear) “5”
Medial meniscus tear (X_10_)	no “0”; yes “1”
Outerbridge grade (X_11_)	no “0”; grade I “1”; grade II “2”; grade III ~ IV “3”
K-L grade (X_12_)	grade 0 “0”; grade I “1”; grade II “2”; grade III ~ IV “3”
Type of surgery (X_13_)	Saucerization “1”; Saucerization with repair “2”; Total meniscectomy “3”
Follow-up time (month)(X_14_)	X_14_ ≤ 60 “1”; 60 < X_14_ ≤ 96; X_14_ > 96
IKDC classification (Y)	< 70 (poor) ~ 70–79 (fair) “1”; 80–89 (good) “2”; ≥90 (excellent) “3”

*BMI* body mass index, *K-L grade* Kellgren–Lawrence grade, *DLM* discoid lateral meniscus, *IKDC* International Knee Documentation Committee

### Statistical method

The data were statistically analysed using SPSS 25.0. A normality test revealed that the measurement data were not normally distributed. The measurement data and enumeration data are described by the median (M) and interquartile range (IQR) and by the number of cases (percentage), respectively. The differences between the preoperative and postoperative knee function scores were analysed using the Wilcoxon rank-sum test. In the univariate analysis, the Mann-Whitney U test and the Kruskall-Wallis rank sum test were used to analyse the differences in rank data between two groups and among multiple groups, severally. The model meets the parallelism through parallel test, and multivariate analysis was conducted using the ordinal logistic regression. *P* < 0.05 was considered statistically significant.

## Results

### General characteristics of the subjects

According to the inclusion and exclusion criteria, 502 patients were eventually included in our study, including 353 females (70.3%) and 149 males (29.7%), 252 (50.2%) left knees and 250 (49.8%) right knees. The median age of onset and the median duration of symptoms were 32.0 years (range, 3 ~ 80 years; IQR, 26.3) and 10.0 months (range, 0.05 ~ 246 months; IQR, 21.0), respectively. Other baseline information on the patients is shown in Table 1. The median postoperative IKDC score of (87.4; range, 41.4 ~ 97.7; IQR, 14.6) was higher than the median preoperative score (57.6; range, 26.9 ~ 64.9; IQR, 9.7) (*P* < 0.001). (Table 3). In the follow-up, none of the patients required reoperation or experienced complications after surgery.

**Table 3:** Preoperative and postoperative IKDC score

	Preoperative M (IQR)/N (%) †	Postoperative M (IQR)/N (%) †	Z	p
IKDC score	57.6 (9.7)	87.4 (14.6)	−19.420	0.000*
< 70 (poor)	502 (100.0%)	55 (11.0%)		
70 ~ 79 (fair)	0(0.0%)	84 (16.7%)		
80 ~ 89 (good)	0(0.0%)	164 (32.7%)		
≥ 90 (excellent)	0(0.0%)	199 (39.6%)		

*IKDC* International Knee Documentation Committee, † *M* median, *IQR* interquartile range, † *N* number

*Statistically significant (*P* < .05)

### Univariate analysis of the correlation between the investigated factors and IKDC classification

Factors such as sex, BMI, work intensity, trauma history, age of onset, symptoms duration, medial meniscus tear, Outerbridge grade, K-L grade and type of surgery were associated with the IKDC classification (*P* < 0.05). Involved knee joint (left or right), Watanabe type, O’Connor tear type and follow-up time did not correlate with the IKDC classification (*P* > 0.05). Table 4.

**Table 4:** Univariate analysis of the correlation between the research factors and IKDC classification

Variable	IKDC grade			Z/kruskall-wallis χ2	P
	Fair and poor N (%) †	Good N (%) †	Excellent N (%) †		
**Sex**				−4.362	0.000*
Female	114(32.3)	119(33.7)	120(34.0)		
male	25(16.8)	45(30.2)	79(53.0)		
**BMI** (kg/m^2^)				66.642	0.000*
< 18.5	7(13.0)	4(7.4)	43(79.6)		
18.5 ~ 25	86(23.8)	129(35.6)	147(40.6)		
≥ 25	46(53.5)	31(36.0)	9(10.5)		
**Work intensity**				74.092	0.000*
Grade 0 ~ 1	39(16.3)	56(23.4)	144(60.3)		
Grade 2	90(37.5)	98(40.8)	52(21.7)		
Grade 3 ~ 4	10(43.5)	10(43.5)	3(13.0)		
**History of trauma**				−2.618	0.009*
No	113(31.4)	113(31.4)	134(37.2)		
Yes	26(18.3)	51(35.9)	65(45.8)		
**Involved knee joint**				−0.843	0.399
Left	65(25.8)	84(33.3)	103(40.9)		
Right	74(29.6)	80(32.0)	96(38.4)		
**Age of onset** (year)				188.575	0.000*
≤ 14	2(2.3)	11(12.5)	75(85.2)		
14 ~ 25	9(8.9)	22(21.8)	70(69.3)		
25 ~ 45	62(30.7)	94(46.5)	46(22.8)		
≥ 45	66(59.5)	37(33.3)	8(7.2)		
**Symptoms duration** (month)				26.939	0.000*
≤ 1	13(20.3)	21(32.8)	30(46.9)		
1 ~ 6	34(22.8)	52(34.9)	63(42.3)		
6 ~ 24	43(23.4)	58(31.5)	83(45.1)		
>24	49(46.7)	33(31.4)	23(21.9)		
**Watanabe type**				−1.867	0.062
Complete	111(26.2)	138(32.6)	174(41.1)		
Incomplete	28(35.4)	26(32.9)	25(31.6)		
**O’Connor tear type**				8.129	0.149
No tearing	12(31.6)	11(28.9)	15(39.5)		
Longitudinal (/bucket handle) tear	50(29.2)	51(29.8)	70(40.9)		
Horizontal tear	31(20.1)	53(34.4)	70(45.5)		
Oblique tear	3(30.0)	4(40.0)	3(30.0)		
Transverse (/radial) tear	15(33.3)	14(31.1)	16(35.6)		
Variant tear	28(33.3)	31(36.9)	25(29.8)		
**Medial meniscus tear**				−2.876	0.004*
No	127(26.2)	162(33.5)	195(40.3)		
Yes	12(66.7)	2(11.1)	4(22.2)		
**Outerbridge grade**				116.370	0.000*
No injury	51(14.2)	129(36.0)	178(49.7)		
Grade I	11(37.9)	6(20.7)	12(41.4)		
Grade II	29(55.8)	18(34.6)	5(9.6)		
Grade III ~ IV	48(76.2)	11(17.5)	4(6.3)		
**K-L grade**				116.570	0.000*
Grade 0	57(15.2)	133(35.5)	185(49.3)		
Grade I	26(46.4)	19(33.9)	11(19.6)		
Grade II	34(77.3)	9(20.5)	1(2.3)		
Grade III ~ IV	22(81.5)	3(11.1)	2(7.4)		
**Follow-up time** (month)				5.542	0.063
≤ 60	47(34.8)	44(32.6)	44(32.6)		
60–96	65(25.6)	81(31.9)	108(42.5)		
>96	27(23.9)	39(34.5)	47(41.6)		
**Type of surgery**				77.186	0.000*
Saucerization	80(19.5)	142(34.6)	188(45.9)		
Saucerization with repair	5(31.3)	5(31.3)	6(37.5)		
Total meniscectomy	54(71.1)	17(22.4)	5(6.6)		

*IKDC* International Knee Documentation Committee, *BMI* body mass index, *K-L grade* Kellgren–Lawrence grade, †*N* number

*Statistically significant (*P* < .05)

### Multivariate analysis of the correlation between the investigated factors and IKDC classification

Male sex was associated with higher IKDC score (*P* = 0.023, odds ratio (OR) = 1.702, 95% confidence interval (CI):1.076–2.697). Compared with BMI ≥ 25 kg/m^2^, BMI < 18.5 kg/m^2^ was associated with better IKDC score (*P* = 0.026, OR = 3.016, 95% CI: 1.138–7.996), while no difference was found between 18.5 kg/m^2^ ≤ BMI < 25 kg/m^2^ and BMI ≥ 25 kg/m^2^ regarding IKDC score (*P* = 0.063, OR = 1.701, 95% CI: 0.972–2.974). In contrast to age of onset ≥45 years, *≤14 years* (*P* = 0.000, OR = 20.780, 95% CI: 7.822–55.147) and *14 ~ 25 years* (*P =* 0.000, OR = 8.516, 95% CI: 3.755–19.317) were predictive of obtaining a good IKDC score, whereas there was no significant difference between the IKDC score of *25 ~ 45 years* and *≥ 45 years* (*P* = 0.098, OR = 1.725, 95% CI: 0.904–3.294). In comparison with symptoms duration> 24 months, the odds of better postoperative IKDC score for symptoms duration *≤1 month* (*P* = 0.001, OR = 3.511, 95% CI: 1.699–7.265), *1 ~ 6 months* (*P* < 0.001, OR = 3.463; 95% CI: 1.914–6.265) and *6 ~ 24 months* (*P* < 0.001, OR = 3.254; 95% CI: 1.855–5.703) were significantly elevated. Compared to the Outerbridge grade III ~ IV, no injury (*P <* 0.001, OR = 6.379; 95% CI:2.545–15.975) and Outerbridge grade I (*P* = 0.01, OR = 4.332; 95% CI:1.142–13.277) were favourable factors for acquiring higher IKDC score, while no difference was found between grade II and grade III ~ IV in terms of IKDC score (*P* = 0.12, OR = 2.134; 95% CI:0.820–5.551). Work intensity, history of trauma, medial meniscus tear, K-L grade and type of surgery were not significant predictors of IKDC score (*P* > 0.05 for all). (Table 5).

**Table 5:** Multivariate analysis of the correlation between research factors and IKDC classification

Variable	B	S.E.	Wald	p	OR	95% CI	
						Lower	Upper
**Sex**							
Male	0.532	0.234	5.151	0.023*	1.702	1.076	2.697
Female‡	–	–	–	–	1.000	–	–
**BMI** (kg/m^2^)							
< 18.5	1.104	0.497	4.926	0.026*	3.016	1.138	7.996
18.5 ~ 25	0.531	0.285	3.467	0.063	1.701	0.972	2.974
≥ 25‡	–	–	–	–	1.000	–	–
**Work intensity**							
Grade 0 ~ 1	0.331	0.506	0.427	0.514	1.392	0.516	3.751
Grade 2	−0.223	0.481	0.214	0.644	0.800	0.312	2.056
Grade 3 ~ 4‡	–	–	–	–	1.000	–	–
**History of trauma**							
No	−0.179	0.232	0.597	0.440	0.836	0.531	1.317
Yes‡	–	–	–	–	1.000	–	–
**Age of onset** (year)							
≤ 14	3.034	0.498	37.069	0.000*	20.780	7.822	55.147
14 ~ 25	2.142	0.418	26.293	0.000*	8.516	3.755	19.317
25 ~ 45	0.545	0.33	2.732	0.098	1.725	0.904	3.294
≥ 45‡	–	–	–	–	1.000	–	–
**Symptoms duration** (month)							
≤ 1	1.256	0.37	11.501	0.001*	3.511	1.699	7.265
1 ~ 6	1.242	0.303	16.826	0.000*	3.463	1.914	6.265
6 ~ 24	1.180	0.287	16.949	0.000*	3.254	1.855	5.703
>24‡	–	–	–	–	1.000	–	–
**Medial meniscus tear**							
No	0.089	0.624	0.021	0.886	1.093	0.322	3.717
Yes‡	–	–	–	–	1.000	–	–
**Outerbridge grade**							
No injury	1.853	0.469	15.629	0.000*	6.379	2.545	15.975
Grade I	1.466	0.572	6.569	0.010*	4.332	1.412	13.277
Grade II	0.758	0.488	2.418	0.120	2.134	0.820	5.551
Grade III ~ IV‡	–	–	–	–	1.000	–	–
**K-L grade**							
Grade 0	0.184	0.690	0.071	0.790	1.202	0.311	4.646
Grade I	−0.112	0.685	0.027	0.870	0.894	0.233	3.425
Grade II	−0.563	0.707	0.634	0.426	0.569	0.142	2.277
Grade III ~ IV‡	–	–	–	–	1.000	–	–
**Type of surgery**							
Saucerization	0.546	0.387	1.983	0.159	1.726	0.807	3.688
Saucerization with repair	−0.262	0.678	0.149	0.699	0.770	0.204	2.907
Total meniscectomy‡	–	–	–	–	1.000	–	–

*IKDC* International Knee Documentation Committee, *BMI* body mass index, *K-L grade* Kellgren–Lawrence grade

‡ represent reference group; *Statistically significant (*P* < .05)

## Discussion

IKDC score, a subjective method of evaluating knee function, not only focuses on the evaluation of the patient’s symptoms and the stability of the knee joint, but also attaches importance to the assessment of knee motor function and is widely used because it allows accurate and comprehensive evaluation of knee function. After analyzing the correlation of 14 independent factors with postoperative IKDC classification, we found that sex, BMI, age of onset, symptoms duration and cartilage injury and its degree affect postoperative IKDC classification, while preoperative work intensity, DLM type, DLM tear and its O’Connor type, combined medial meniscus injury, K-L grade, postoperative follow-up time and type of surgery did not significantly affect the postoperative result.

Male sex is a favourable factor in many orthopaedic diseases, but its correlation with postoperative efficacy in patients with DLM is controversial. Ahn [23] et al. demonstrated that male sex was conducive to good postoperative outcomes by evaluating 260 patients with DLM. Through investigating 502 patients with DLM, we also found that male sex was a protective factor for good knee function (*P* = 0.023, OR = 1.702, 95% CI: 1.076–2.697). Compared with males, on one hand, females have increased rates of cartilage loss and progression of cartilage defects at the knee [24]. On the other hand, the knee articular cartilage volume is smaller and the Q angle is greater in females [13, 25, 26]. Thus, females are more susceptible to cartilage lesions and osteoarthritis [24] and being female is associated with poor postoperative clinical outcomes. However, Chen et al. [12] and Kose et al. [17] found that sex has no significant effect on postoperative result of DLM; this finding may be related to the small sample size in their study (*n* = 39 cases, *n* = 48 cases, respectively).

For symptomatic DLM, numerous studies have found that the earlier the age of onset is, the shorter is the duration of symptoms (especially < 12 months) and that the earlier the surgical intervention is performed, the better is the prognosis [11, 12, 14, 23, 27]. We found that the age of onset < 25 years, especially < 14 years (*P* < 0.001, OR = 37.069; 95% CI: 7.822–55.147), and the symptoms duration < 24 months (*P <* 0.001, OR = 3.254; 95% CI: 1.855–5.703) are advantageous for postoperative efficacy. It has been reported that the younger age of onset and a shorter course of the disease are correlated with a lower risk of articular chondromalacia and damage caused by DLM lesions [11, 13, 28]. Moreover, early normalization of DLM morphology by surgery not only increases the mobility of the meniscus, but also enhances the adaptability of the meniscus to the tibiofemoral surface, thereby reducing damage and degeneration of the meniscus and articular cartilage arising from excessive stress concentration [11, 14, 29]. In addition, shaping DLM in childhood may improve the dysplasia of the femoral condyle and the abnormality of the lower limb alignment, thus abating the risk of cartilage degeneration and delaying the occurrence and development of osteoarthritis [16, 30, 31].

This study found that BMI < 18.5 kg/m^2^ is associated with a higher likelihood of obtaining better postoperative results (*P* = 0.026, OR = 3.016; 95% CI: 1.138–7.996). Fu [13] et al. found that patients with BMI > 23.0 kg/m^2^ were more likely to suffer from articular cartilage lesions. High BMI has been shown to be the main risk factor for knee osteoarthritis, as obesity can lead to excessive compression of the meniscus and to loss of and pathological changes in the articular cartilage [32–34]. Hence, lower BMI is related to lower occurrence of articular cartilage lesions and knee osteoarthritis and thus better postoperative efficacy. Nonetheless, we found no effect of work intensity on the postoperative outcomes of symptomatic DLM (*P* > 0.05); this may be because that work intensity is more reflects the activity of entire body rather than the pressure on the meniscus and cartilage of the knee.

The results of this study indicate that the absence of articular cartilage lesions (*P* < 0.001, OR = 6.379; 95% CI:2.545–15.975) and Outerbridge grade I (*P* = 0.001, OR = 4.322; 95% CI: 1.412–13.277) are beneficial factors for postoperative recovery of knee function. Outerbridge grade ≥ II is an unfavourable factor for postoperative outcome (*P* = 0.12, OR = 2.134). The clinical manifestations of an articular cartilage lesion may not be obvious in the short term, but most patients will eventually have knee degeneration associated with cartilage damage, which leads to irreversible severe osteoarthritis drastically affecting knee function [35]. Hiroshi et al. [36] consider that the Outerbridge grade can directly reflects the severity of the cartilage lesions and is the decisive factor affecting the long-term outcomes after meniscal surgery. Although K-L grade is an imaging index that is used to assess the severity of degeneration of the knee, we observed no relationship between KL grade and postoperative outcome, probably because X-rays are less sensitive in visualizing cartilage lesions [37] and because early joint space reduction is secondary not to articular cartilage thinning but to meniscal compression [14]. Moreover, Kose et al. [17] have shown that the combined medial meniscus tears did not affect postoperative outcomes, which is similar to the result of our study.

Generally, surgical methods for the arthroscopic treatment of symptomatic DLM by arthroscope includes saucerization (partial meniscectomy), saucerization with repair and total meniscectomy [27, 28, 38–40]. At present, the effect of surgical mode on postoperative outcomes is disputable. Ahn et al. [41] considered that partial meniscectomy with repair has a good efficacy in children with symptomatic DLM compared with total meniscectomy; this may be because partial meniscectomy with repair can prevent early degenerative changes of the joint [2]. However, Lee et al. [11] harbour the opposite opinion that residual discoid meniscus tissue is prone to degeneration and re-injury due to its abnormally fibrous structure, which may lead to adverse clinical effects. Considering the high cost and uncertain effectiveness of repair, repair of the abnormal anatomy in a torn DLM is not recommended [42]. Some scholars have not found the differences in clinical outcomes between partial meniscectomy and total meniscectomy in the short or medium term, but the clinical efficacy of partial meniscectomy is better than that of total meniscectomy in the long-term follow-up [28, 31, 38–40, 42–44]. Conversely, Ikeuchi et al. [5] reported that the results of partial meniscectomy were significantly worse than those of total meniscectomy. However, Lee et al. [45] and Wong and Wang [27] concluded that there was no significant difference among these three surgical methods in postoperative outcomes. In addition, a systematic review did not find a difference in postoperative outcome between partial meniscectomy with repair and saucerization, but these two methods have significantly improved outcomes over total meniscectomy [42]. In the present study, we discovered that the type of surgery does not affect the postoperative result. The discrepancies in the results obtained in these studies may exist because the choice of surgical method was affected by factors such as age at surgery, location of DLM tear, severity of the DLM tear and other factors and because the number of patients who underwent saucerization with repair and total meniscectomy were small.

Similar to the results of other studies [11, 12, 17], we also found that DLM type has no significant effect on postoperative efficacy. This may be why no significant differences were found in the incidence of articular cartilage lesions and postoperative discoid meniscus morphology among different types of discoid meniscus [13, 29, 46].

Regarding DLM tear, some studies reported that DLM tears could lead to degeneration of the articular cartilage and osteoarthritis in the long term [29, 47], thus contributing to poor postoperative outcomes. However, Ding et al. [29] and Kose et al. [17] concluded that discoid meniscus injuries are not correlated with articular cartilage lesions. In our study, DLM tears did not affect the postoperative effect, and no difference in cartilage damage was observed during arthroscopy with respect to whether or not DLM tear exist; this may be because that the majority of our patients with DLM tears had obvious symptoms and received timely diagnosis and treatment.

Concerning the influence of O’Connor tear type on the postoperative result, Chen et al. [12] and Badlani et al. [48] considered that radial tears lead to poor postoperative outcomes. Ahn et al. [28] found that compared with other tear types, the duration of symptoms of horizontal meniscus tear, as a degenerative tear, is longer and the postoperative residual meniscus tissue is reduced and fragile, which may accelerate the radiological progression of postoperative KL grade 3/4 osteoarthritis. However, other studies observed that the duration of symptoms in cases of horizontal tear may not be significantly different from that in cases with other tear types [2, 49]. Here, we did not find a correlation of O’Connor tear type with postoperative effect; this may be attributed to the fact that the severity of cartilage damage is not related to the type of DLM tear [13] as well as to the fact that the difference in thickness between DLM and normal meniscus is not obvious even though removing a layer of horizontal meniscus tear as the discoid meniscus is thicker than the normal meniscus.

Longer follow-up is believed to be associated with more severe articular cartilage degeneration and clinical symptoms and worse knee joint function [11, 44]. In our study, the median follow-up time was 75.4 (range, 41 ~ 123; IQR, 33.7) months, and the final follow-up time was not correlated with postoperative efficacy of symptomatic DLM; this may be due to the small number of patients with follow-up time over 120 months.

We acknowledge that our study has some limitations. At the final follow-up, the assessment of postoperative efficacy didn’t analyse the imaging changes but only evaluated the subjective functional parameters; thus, there was no objective evaluation index that corresponded to the postoperative outcome. Moreover, this study is only a retrospective multivariate analysis, and the conclusions obtained should be further confirmed by prospective studies.

## Conclusion

Arthroscopic treatment of symptomatic DLM is safe and effective, but its clinical efficacy is affected by many factors. Specifically, male sex, BMI < 18.5 kg/m^2^, age of onset < 25 years (especially < 14 years) and symptoms duration < 24 months are conducive to good postoperative outcomes. Combined articular cartilage injury (Outerbridge grade ≥ 2) is more likely to lead to poor postoperative effect. However, complicating medial meniscus injury, K-L grade, preoperative work intensity, DLM type, DLM tear and its O’Connor type, type of surgery, and postoperative follow-up time did not significantly affect the postoperative result.

## Data Availability

The patient’s personal information and imaging data obtained at following-up were stored on disc. The data are available from the corresponding author upon request. G. Chen should be contacted with requests for data and materials.

## Abbreviations

DLM: Discoid lateral meniscus
BMI: Body mass index
IKDC: International Knee Documentation Committee
K-L: Kellgren-Lawrence
MRI: Magnetic resonance imaging
M: Median
OR: Odds ratio
IQR: Interquartile range
CI: Confidence interval
